# Flipped Classrooms in Medical Education: Improving Learning Outcomes and Engaging Students in Critical Thinking Skills

**DOI:** 10.7759/cureus.48199

**Published:** 2023-11-03

**Authors:** Adwait Nichat, Ujwal Gajbe, Nandkishor J Bankar, Brij Raj Singh, Ankit K Badge

**Affiliations:** 1 Medical Education, Datta Meghe Medical College, Datta Meghe Institute of Higher Education and Research (Deemed to be University), Nagpur, IND; 2 Anatomy, Datta Meghe Medical College, Datta Meghe Institute of Higher Education and Research (Deemed to be University), Nagpur, IND; 3 Microbiology, Jawaharlal Nehru Medical College, Datta Meghe Institute of Higher Education and Research (Deemed to be University), Wardha, IND; 4 Microbiology, Datta Meghe Medical College, Datta Meghe Institute of Higher Education and Research (Deemed to be University), Nagpur, IND

**Keywords:** flipped classroom, problem-based learning, competency-based medical education, medical education, traditional teaching

## Abstract

The flipped classroom (FC) model involves students independently acquiring knowledge before in-person class sessions, during which they engage in active discussions and problem-solving. Various methods to implement FC are quizzes, e-content, case-based learning, problem-based learning, and reading assignments. The advantages of the FC approach included improved student preparation, active participation, and the promotion of critical thinking skills. Some disadvantages identified are technical problems like internet connection, improper planning and preparation, which increases teacher workload, and lack of self-motivation. This review underscores the potential of the FC approach to improve medical education by promoting independent learning, active participation, and deeper understanding. Consideration of factors such as curriculum design, faculty development, technological infrastructure, and student readiness is vital for successfully implementing the FC model. Balancing self-directed study with meaningful face-to-face interactions remains crucial to harnessing the full benefits of this innovative approach. By leveraging technology and student-centered methods, medical educators can create an enriched learning experience that positively influences future healthcare professionals.

## Introduction and background

Medical education refers to teaching programs designed to serve the community in the near future. Good role models and learning environments which are examples of professional and organizational behaviors to be adopted, learning through practice, simulation programs, and educational tools such as electronic learning (e-learning) systems, good assessment and feedback systems, and portfolios that demonstrate and discuss professional progress are key elements of medical education programs [1]. Several advantages of traditional teaching involve face-to-face interactions between students and teachers. Face-to-face interactions provide a supportive learning environment with a positive psychological impact and motivate even less motivated students to participate [2]. Competency-based medical education (CBME) is a standardized framework for measuring student performance, focusing on the key learning components of good clinical practice. It also measures learning outcomes in training programs based on self-assessment, objective assessment, and multi-source assessment. It can be used for training in all medical fields [3,4]. One of the goals of CBME is self-directed learning, and flipped classroom (FC) is based on this concept, making FC an integral part of the CBME curriculum [5,6]. The main objective of CBME is to create competent Indian medical graduates (IMG) using a skill-based approach while also providing them with metacognition skills [3, 7]. The objective of this review is to explore the effectiveness of FC in medical education.

## Review

Methods 

To conduct a comprehensive literature search, we used the PubMed and Google Scholar search. We searched for articles published between 2018 and 2023 using the following search terms: (Flipped classroom) OR (flipped classroom) AND (problem-based learning) AND (case-based learning) OR (virtual classroom) AND (traditional teaching). We applied the following inclusion criteria for the final review: (1) English language, (2) relevant to FC in medical education, (3) full text available, and (4) published in specified time frame.

Articles Screened

After conducting the initial search, we identified a total of (n=726) articles across the searched databases. We conducted an initial screening of titles and abstracts, which excluded (n=267) articles. After full-text screening of a total of (n=403) articles, we excluded (n=234) articles for not being retrieved. After screening (n=169) articles for eligibility, we excluded (n=150) articles that were not related to the topic and not in the English language leaving a total of (n=19) articles.

Duration and Number of Articles Included in the Final Review

**Figure 1:**
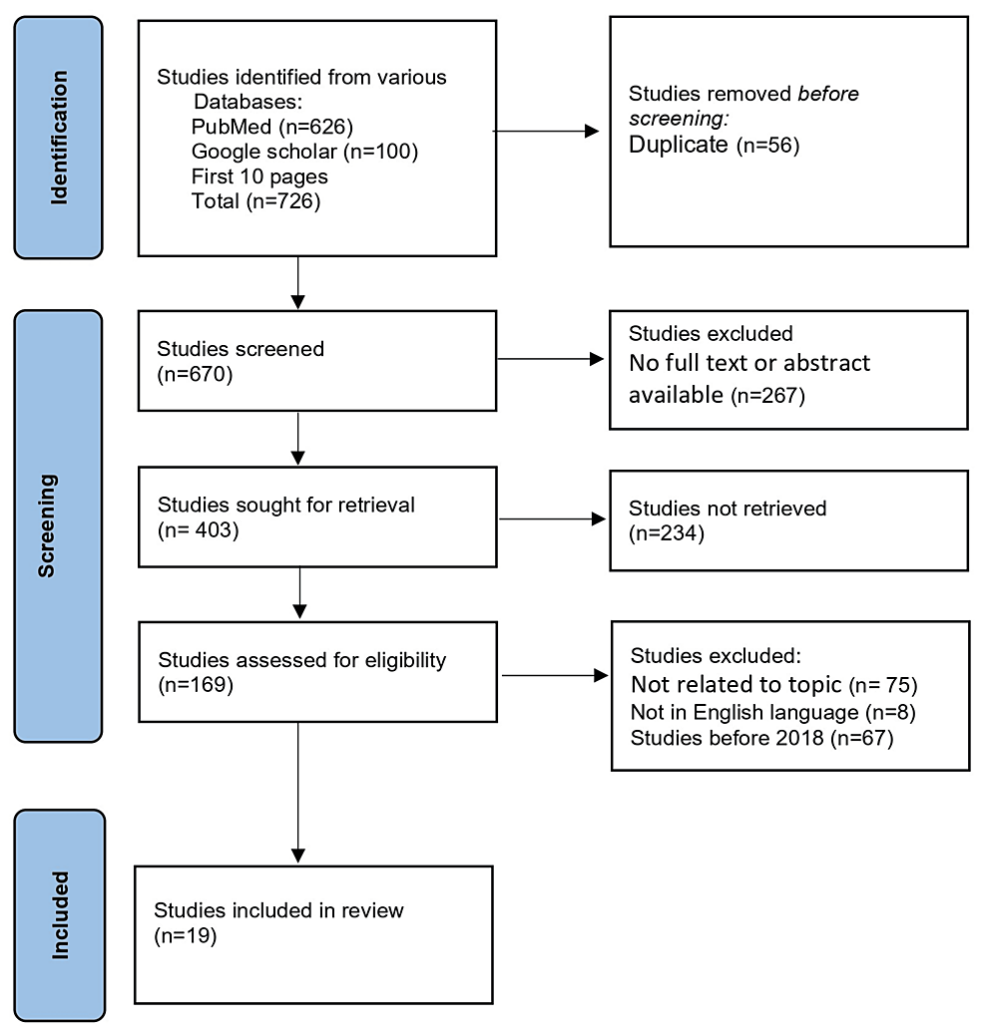
PRISMA flowchart n: number of studies; PRISMA: Preferred Reporting Items for Systematic Reviews and Meta-Analysis

The literature search was conducted in August 2023. The final review included a total of 19 articles from 2018 to 2023 (Figure 1).

The articles included in the review were each FC based on micro-video courses, self-learning-based online videos, texts, and traditional methods (Table 1).

**Table 1:** Articles included in the study OSCE: objective structured clinical examination; FC: flipped classroom; NPE: near-peer education; NBME: National Board of Medical Examiners; ACLS: advanced cardiopulmonary life support

Sr no	Authors	Year	Methods	Methods of assessment	Findings
1	Ji et al. [7]	2022	One group was given video links and resource materials prior to classes, while the other group was given traditional teaching	Physiology final exam papers	FC has a positive impact on students' learning in physiology courses without affecting the learning of other courses
2	Wu et al. [8]	2022	One group was given micro-video lectures along with theory lectures, while the other group was given only theory lectures	Theory test	Application of FC by using micro-video lectures proves to be effective in teaching pharmacology to medical students
3	Sourg et al. [9]	2023	One group was given reading resources and video lectures two days before class, while the other group received formal lecture	Pre-test and post-test results	There was no significant difference between the pre-test and post-test results of the two groups
4	Golaki et al. [10]	2022	Presentation and software were used to prepare the content for students of one group which was uploaded two weeks prior on the website, while the other group was taught by conventional method using workshop	Pre-test and post-test results	FC along with NPE proved to be beneficial for nursing students
5	Wang et al. [11]	2021	One group was given videos and other resources via e-learning system, while lecture session was given to the other group	A multilevel linear regression analysis	FC proved to be effective in acquiring knowledge
6	Paul et al. [12]	2023	One group was given only online instructions, while the other group was given both online and in-person instructions	Peer-reviewed clinical reasoning exam, NBME scores, and OSCE	There was no difference observed although student satisfaction was high in both methods
7	Lu et al. [13]	2023	One group was given a self-study task list, while the other group received traditional lectures	Final exam scores	FC helps in improving learning outcomes
8	Yang et al. [14]	2020	One group was assigned to do web-based learning and group discussion before class, while lecture was given to the other group	Pre-quiz and post-quiz scores	FC along with web-based learning improves students' interest and thinking ability
9	Beom et al. [15]	2018	One group was given PowerPoint along with explanation beforehand, while the other group's lesson was explained using PowerPoint in class	Simulation rating scores and students' satisfaction survey	ACLS results showed that there was no significant difference between traditional teaching and FC
10	Wen et al. [16]	2022	One group was provided with videos and reference books, while the other group was subjected to traditional teaching	Pre-test and post-test results	FC proved to improve students' interpretation ability and their enthusiasm towards learning
11	Zhong et al. [17]	2022	One group was given e-content prior to class, while the other group was given theory lectures	Pre-test and post-test results	FC accompanied with peer-peer interaction improved knowledge learning
12	Lu et al. [18]	2021	One group was given pre-class video lectures before in-class-based case-based learning, while the other group was given reading assignments and in-person lecture	Pre test, post test, and three-month retention	Students gave positive feedback on FC
13	Dombrowski et al. [19]	2018	One group was provided with e-learning before practical, while the other group was directly taught practical	Theory test	FC helped to save time for introducing new topics
14	McCall et al. [20]	2021	One group was provided access to online lecture along with classes, while the other group was taught using traditional method	Pre-test and post-test results	FC proved to be beneficial
15	Heitmann et al. [21]	2022	One group was given class material beforehand, while the other group was taught using traditional teaching	School grades	FC helps to motivate students to study
16	Shikino et al. [22]	2022	One group was given pre-class study material, while the other group was subjected to traditional teaching	Post-training questionnaire	FC proved to be effective
17	Ng et al. [23]	2023	One group was given online micro-modules, while the other group was given didactic lectures	Pre-intervention and post-intervention multiple-choice questions	Application of FC by means of micro-modules can help in replacing didactic lectures
18	Blanie et al. [24]	2022	One group was provided with theory notes beforehand, while the other group was directly subjected to training session	Composite learning score	No significant difference in acquisition of training skills was found
19	He et al. [25]	2019	One group was given micro-lectures before class, while the other group was directly given theory lectures	Teacher-student interaction and questionnaires	FC improved students' score and also helped in developing positive attitude within students

Nine studies [9,10,14,16,17,18,20,22,23] conducted pre tests and post tests. In each study [9,10,14,16,17,18,20,22,23], students were divided into two groups: one group was subjected to traditional teaching, while the other group was subjected to various methods to implement FC which were reading resources and video lectures [9,16,18,20,22,23], presentation [10], web-based learning [14], and e-content [17]. All studies [9,10,14,16,17,18,20,22,23] observed that FC is an effective tool. One study [25] divided students into two groups: one group was given micro-video lectures before class, while the other group was directly given theory lectures. Teacher-student interaction and questionnaires were used to assess the students, and it found that the FC model improved student performance. Six studies [7,8,13,15,19,21] assessed students based on final exam scores. In each study, students were divided into two groups: one group was subjected to traditional teaching, while for the other group, various methods were used including videos and resource materials [7,8,19,21], self-study [13], and PowerPoint [15]. Five studies [7,8,13,19,21] observed that FC is an effective tool for improving students' performance, while one study [15] did not observe any change in the students' performance. One study [11] conducted multilevel regression and observed that FC is an effective tool. In this study, the students were divided into two groups: one group was given videos and other resources via e-learning system, while lecture session was given to the other group. One study [12] observed the results based on OSCE (objective structured clinical examination) and NBME (National Board of Medical Examiners) scores and did not observe any change in students' performance. In this study, students were divided into two groups: one group was given only online instructions, while the other group was given both online and in-person instructions. One study [24] observed the result based on the composite learning score and found no significant difference. In this study, students were divided into two groups: one group was provided with theory notes beforehand, while the other group was directly subjected to training session.

Medical education

The systematic process of preparing interested and qualified people to become doctors is known as medical education. The Bachelor of Medicine, Bachelor of Surgery (MBBS) degree is considered capable of handling the responsibilities of a physician of first contact like patient care, medical practice, administrative duties, and ethical and legal duties [26]. The main objective of the National Medical Commission (NMC) project is to ensure that IMG are capable of serving as primary care physicians in their communities. The NMC project aims to improve the quality of medical education in India and enhance the practical skills of IMG. The NMC has taken an important initiative to introduce CBME to the undergraduate medical curriculum in India. The NMC clearly defined the competencies that an undergraduate medical student must have to become a globally competent IMG. The regulatory body has made significant efforts to design programs with the expert team and has also planned "training of trainers" from faculty at medical colleges throughout India, through the Curriculum Implementation Support Program (CISP) I and II, an implementation support program for schools across India [27]. Additionally, the project also seeks to bridge the gap between theoretical knowledge and hands-on experience, allowing IMG to confidently handle diverse medical cases and contribute to the overall development of the healthcare system [26,28]. The learning results and the competency of medical graduates are substantially impacted by the attitudes of both teachers and students. Medical educators' professionalism, management, and leadership abilities may be enhanced by well-crafted faculty development programs, which will help students become competent doctors [26,29]. Faculty members are primarily responsible for carrying out this significant duty. They are the most valuable resources and the foundation of any higher education institution. The role of the facilitator is to pay appropriate attention to the fields of competence, management, and leadership and to make accurate and comprehensive planning for students to become qualified future doctors in the role of therapists, managers, teachers, supporters, and researchers [30].

FC

**Figure 2:**
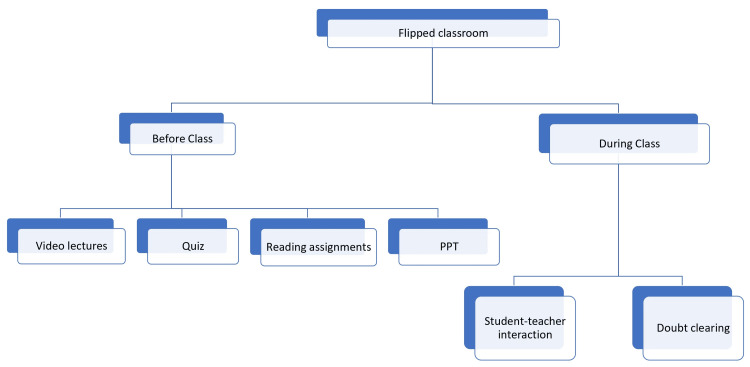
Concept of flipped classroom PPT: PowerPoint presentation References: [9,31-40]

FC is a technique where knowledge is acquired independently by a student prior to a classroom encounter. This knowledge is then applied during in-person interactions taken by a teacher, often in the form of case-based discussions, helping to achieve higher-level problem-solving. FC is an effective way to promote active learning and critical thinking skills among students. Having students acquire knowledge independently before class makes them better prepared to engage in meaningful discussions and analyze real-life scenarios. This approach enhances problem-solving abilities and encourages independent learning and self-motivation. FC provides a valuable framework for bridging the gap between theory and practice in classrooms [31-33]. In a traditional face-to-face learning environment, fundamental concepts can be supplemented with online or asynchronous activities [31,34]. By using various forms of technology to share lecture materials outside of the classroom and with greater student-teacher interactions inside the classroom, FC focuses on student-centered learning rather than teacher-centered learning [9,35]. It is an inverted method of instruction that disseminates lecture materials outside of the classroom using videos, podcasts, or slides [36,37]. It can improve student learning efficiency and deepen student understanding, but teachers may lose the constraints on students [36,38]. In the field of medical education, FC serves as an excellent resource and is suitable for students so that they can participate more actively and focus on class interaction while using the pre-class time to acquire a lot of knowledge in their leisure time. The FC model allows students to watch pre-recorded lectures or read assigned materials before class. This way, students can grasp the foundational concepts at their own pace and have more time for critical thinking and problem-solving during in-person sessions. Additionally, FC promotes self-directed learning and encourages students to take ownership of their education, resulting in a deeper understanding and retention of the material [9,39,40]. Figure 2 shows the concept of FC.

Various methods used for implementing FC

E-Content

Video is the most common type of e-content which can be viewed anytime and at a desired pace [41]. Video-based learning provides an avenue to tackle a lot of educational issues. As most of the people have mobile phones and access to the internet, video lectures can help deliver lectures more easily [42]. Virtual reality helps in improving students' understanding of the topic [43] and is emerging as a new technique for presenting simulation [44].

Quiz

Medical quizzes often follow one of the two formats: case-based or image-based. This method aids in bridging the knowledge gap between standard classroom instruction and clinical application. The quiz is a simple tool that enhances didactic lectures by helping students learn and understand more. Being an interactive tool centered on students, it promotes regular feedback mechanisms and encourages active student participation. Web-based quiz games can also be used to summarize the key content [45].

Team-Based Learning (TBL) and Case-Based Learning (CBL)

The pedagogies of CBL and TBL share characteristics such as the use of a real clinical case, active small group learning, activation of prior knowledge, and application of newly learned knowledge. In CBL, teachers guide students as they apply new knowledge to these real-world clinical issues and engage in peer learning. Unlike problem-based learning (PBL), which is intended to allow teachers to criticize and guide students, CBL promotes an organized and critical approach to clinical problem-solving. CBL also encourages students to work collaboratively, fostering teamwork and communication skills essential in the medical field. The emphasis on real-world cases in CBL helps students develop a deeper understanding of how theoretical concepts apply to practical situations [46,47]. TBL provides active and structured small group learning methods and can be applied to large-scale classes. Students' responsibility is achieved through specific TBL steps, including preparatory preparation, preparation assurance tests, problem-solving activities, and immediate feedback [48].

Reading Assignments

Students should be provided with pre-class reading materials such as handouts or worksheets, instructor-developed texts, or other reading materials. They can also be assigned to read specific chapters or sections from textbooks or articles related to the topic. Research papers and scholarly articles help in promoting critical thinking among students. This approach allows students to engage with the material before coming to class, promoting a deeper understanding of the content. It also encourages independent research and analysis, as students must locate and read additional sources beyond the assigned readings. By incorporating research papers and scholarly articles, students are exposed to expert perspectives and encouraged to evaluate the information presented critically. This enhances their critical thinking skills and fosters a deeper appreciation for the subject matter [49-51].

Advantages and disadvantages of FC

FC helps to improve student engagement and encourages students in developing a deeper understanding of the topic. It helps in learning through projects, activities, and discussion which not only increases peer-peer interaction but also helps students to think out of the box. Knowing that each student has a different pace to acquire knowledge, FC helps students to learn at their own pace and do multiple revisions of the topic. As videos and class notes are provided beforehand to students, in-class time can be utilized for teacher-student interaction and to address students' doubts. FC gives flexibility to students by allowing them to learn anytime and anywhere and also helps to teach students time management and self-discipline [52-54].

Some students may not complete the pre-class assignments, and use of e-content which is not validated, internet issues, and the requirement of special software may cause problems. For the proper implementation of FC, thorough planning and preparation of both teachers and students is required which also increases the workload of teachers. Students may lack the motivation to self-study a topic beforehand or may not understand the topic on their own. Not all topics may be suitable to be taught using FC. Studying alone at home may lead to students feeling isolated or disconnected with the teacher [52-54]. The advantages and disadvantages of FC are listed below (Table 2).

**Table 2:** Advantages and disadvantages of flipped classroom References: [52-54]

Advantages	Disadvantages
Increases student engagement	Some students may not complete pre-class assignments which may lead to confusion during class
Provides flexibility to students allowing them to learn anytime and anywhere	Technical problems such as bad internet connection or the requirement of special software for a smartphone and tablet
Helps in learning through discussion, activities, and projects	Requires planning and proper preparation by the teachers
Students can learn at their own pace and can also do multiple revisions	Some students may lack self-motivation
Provides more time for teacher-student interaction	Increases the workload of teachers
Students learn time management and self-discipline	Not all topics may be suitable for flipped classroom
Encourages a deeper understanding of the topic	Students may feel isolated or disconnected

## Conclusions

The emergence of the FC as a cutting-edge educational strategy holds promise for improving medical students' learning outcomes and experiences. The FC paradigm can benefit medical students' learning outcomes, learner engagement, and critical thinking skills. Careful consideration of variables such as curriculum design, technological infrastructure, faculty development, and student preparation is necessary for its successful adoption. The success of the FC approach depends on striking a balance between independent study and significant face-to-face contact, maximizing the advantages of both elements. Medical educators can continue to create a revolutionary educational experience that benefits both students and the future of healthcare by using technology, active learning, and student-centered techniques.
